# Processing of Pheromone Information in Related Species of Heliothine Moths

**DOI:** 10.3390/insects5040742

**Published:** 2014-10-14

**Authors:** Bente G. Berg, Xin-Cheng Zhao, Guirong Wang

**Affiliations:** 1 Department of Psychology, Norwegian University of Science and Technology, Trondheim 7489, Norway; 2 Department of Entomology, College of Plant Protection, Henan Agricultural University, Zhengzhou 450002, China; E-Mail: xincheng@henau.edu.cn; 3 State Key Laboratory for Biology of Plant Disease and Insect Pests, Institute of Plant Protection, Chinese Academy of Agricultural Sciences, Beijing 100193, China

**Keywords:** pheromone, interspecific signal, macroglomerular complex, odorant receptor, olfactory sensory neuron, compartmentalization of sensory neurons, lateral horn

## Abstract

In heliothine moths, the male-specific olfactory system is activated by a few odor molecules, each of which is associated with an easily identifiable glomerulus in the primary olfactory center of the brain. This arrangement is linked to two well-defined behavioral responses, one ensuring attraction and mating behavior by carrying information about pheromones released by conspecific females and the other inhibition of attraction via signal information emitted from heterospecifics. The chance of comparing the characteristic properties of pheromone receptor proteins, male-specific sensory neurons and macroglomerular complex (MGC)-units in closely-related species is especially intriguing. Here, we review studies on the male-specific olfactory system of heliothine moths with particular emphasis on five closely related species, *i.e.*, *Heliothis virescens*, *Heliothis subflexa*, *Helicoverpa zea*, *Helicoverpa assulta* and *Helicoverpa armigera*.

## 1. Introduction

Male and female moths are able to communicate over remarkably long distances [1]. This sexual interaction is based on a few female-produced molecules being recognized by a distinct neural arrangement possessed by the male. Thus, the male-specific olfactory pathway of moths offers the opportunity to investigate a neural network that is relatively simple and at the same time of fundamental importance for the survival of the species. Among the moths most extensively studied is a handful of the monophyletic subfamily, Heliothinae (Lepidoptera: Noctuidae), consisting of more than 365 species distributed on all five continents [2]. This group is especially interesting due to the fact that sympatric species use female-produced compounds for communication, not only within, but also across, the species. Thus, the male-specific olfactory system of these moths typically includes two functionally distinct arrangements, one carrying pheromone information underlying attraction and sexual behavior and the other signal information emitted from heterospecifics, ensuring that the male orients to the conspecific female and not the heterospecific [3].

Furthermore, an essential reason for using heliothine moths as objects for the exploration of chemosensory mechanisms is their vast damage to food and fiber crops. All major pests of this subfamily are included in the two genera, *Heliothis* and *Helicoverpa* [2,4]. Here, we will review previous studies on coding mechanisms for pheromone and interspecific signal information in five species of heliothine moths, *i.e.*, *Heliothis virescens*, *Heliothis subflexa*, *Helicoverpa zea*, *Helicoverpa assulta* and *Helicoverpa armigera*.

## 2. Female-Produced Compounds Evoke Behavioral Responses in Conspecific and Heterospecific Males

Heliothine females of different species produce pheromone blends partly sharing the same constituents. Gland extracts of the species mentioned above all contain Δ7, Δ9 and Δ11 isomers of hexadecenal, hexadecenyl acetate, hexadecenol and the corresponding saturated analogs [5,6,7,8,9,10,11,12]. In addition, Δ9 tetradecenal and/or tetradecenyl acetate have been found in *H. virescens* and *H. armigera*. In the majority of the species studied, *cis*-11-hexadecenal (Z11-16:AL) is the most abundant pheromone compound. Two species, *H. assulta* and *Helicoverpa gelotopoeon*, produce a different major constituent, *i.e.*, *cis*-9-hexadecenal (Z9-16:AL) and hexadecenal (16:AL), respectively [11,13]. Actually, no Z11-16:AL has been found in the gland extract of *H. gelotopoeon* [13].

In addition to the major component, one other constituent among the totally five-to-nine compounds emitted from the heliothine female is usually necessary as part of the pheromone blend for eliciting sexual behavior in conspecific males (Table 1) [5]. Because several species use the same major component, Z11-16:AL, the species-specificity of the binary pheromone blend is ensured by the presence of a critical secondary component, which to a larger extent varies among the species [10,14,15,16]. Furthermore, the distinctness of the binary blend is provided by the relative abundance of the two components, and in *H. subflexa*, yet a third component, *cis*-11-hexadecenyl acetate (Z11-16:AC), has been found to be essential for optimal male attraction [17].

**Table 1:** Principal pheromone constituents and interspecific compounds detected by heliothine males.

Species		*H. virescens* ^1^	*H. subflexa* ^2^	*H. zea* ^3^	*H. gelotopoeon* ^4^	*H. assulta* ^5^	*H. armigera* ^6^
Pheromones	Primary component	Z11-16:AL	Z11-16:AL	Z11-16:AL	16:AL	Z9-16:AL	Z11-16:AL
	Secondary component(s)	Z9-14:AL	Z9-16:AL Z11-16:OH	Z9-16:AL	Z9-16:AL	Z11-16:AL	Z9-16:AL
	Ratio emitted from the female gland	100:3.9	100:66:40.7	100:1.8	100:84	100:6.7	100:4.5
Antagonists		Z11-16:AC Z11-16:OH	-	Z9-14:AL Z11-16:AC	Z11-16:AL	Z9-14:AL Z9-16:OH	Z9-14:AL Z11-16:OH

References: ^1^ [6,18]; ^2^ [8,17]; ^3^ [7,19,20,21,22]; ^4^ [13]; ^5^ [11,12,23]; ^6^ [9].

Behavioral tests have revealed that some components are used as interspecific signals to avoid heterospecific mating mistakes (Table 1). One example is the interplay between *H. virescens*, *H. zea* and *H. subflexa*, whose adults are sympatric and synchronic in North America. The addition of Z11-16:AC produced by *H. subflexa* females to the pheromone blend of *H. virescens* or *H. zea* has been shown to suppress upwind flight and source location in males of the two latter species [18,19,20]. A similar behaviorally antagonistic effect on males of the two species is reported for *cis*-11-hexadecenol (Z11-16:OH) [14,21,22]. Another compound frequently reported as an interspecific signal is *cis*-9-tetradecenal (Z9-14:AL). This particular component is found to suppress sexual attraction in *H. zea* [23] and the two Asian species, *H. armigera* and *H. assulta* [9,24]. The behaviorally inhibiting effect of Z9-14:AL is explained by the fact that the American *H. virescens* and the Asian *H. peltigera*, which are sympatric with the above mentioned species in the respective continents, both utilize Z9-14:AL as the second principal constituent of their respective pheromone blends [5,25].

## 3. Peripheral Arrangement of the Male-Specific Olfactory Pathway

**Figure 1:**
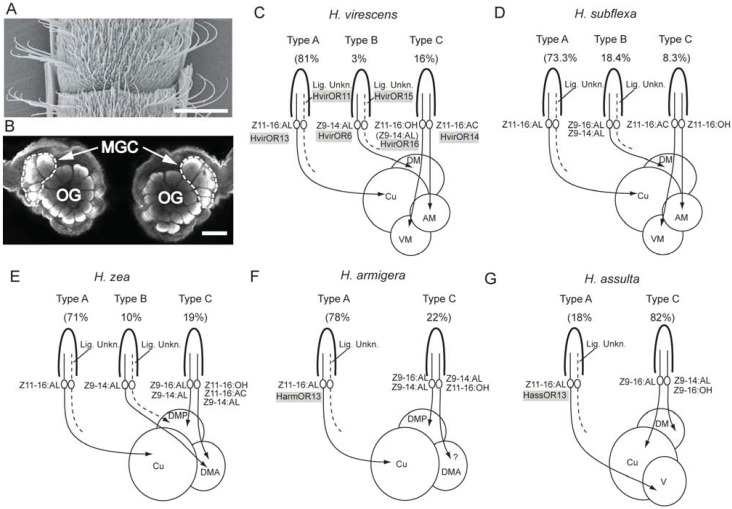
Overview of the first level of the male-specific olfactory pathway in the five most extensively studied species, *Heliothis virescens*, *Heliothis subflexa*, *Helicoverpa zea*, *Helicoverpa armigera* and *Helicoverpa assulta*. (**A**) Scanning electron micrograph of the trichoid sensilla of the *H. virescens* male, adapted from Baker *et al*. [29]. Scale bar: 100 µm. (**B**) Confocal image showing the antennal lobe of a *H. virescens* male, including the macroglomerular complex (MGC) and the more numerous ordinary glomeruli (OG). Scale bar: 100 µm. (**C**–**G**) Schematic drawing of the male-specific sensory axons present in five heliothine species, adapted from Lee *et al*. [35]. The drawing demonstrates, for each species: (1) the organization of sensory neurons within distinct sensillum types; (2) the sensory axons’ termination region within the MGC; and (3) some of the pheromone receptors identified in the various species, *i.e.*, those assumed to be associated with particular physiological categories of male-specific sensory neurons. The relative abundance of each sensillum type is stated in parenthesis. AM, anteromedial glomerulus; Cu, cumulus; DM, dorsomedial glomerulus; DMA, anterior dorsomedial glomerulus; DMP, posterior dorsomedial glomerulus; V, ventral glomerulus; VM, ventromedial glomerulus; Lig. Unkn., ligand unknown.

As in other moths, the antennae of heliothine males are equipped with a large number of olfactory sensory neurons (OSNs) specifically tuned to female-produced compounds. These male-specific neurons are housed inside long curved hairs, sensilla trichodea [26], being arranged in sequences of three to four circumferential rows on the flagellum segments and in such a way that the longest hairs are located adjacent to the scaled side of the antenna (Figure 1A), [27,28,29]. Generally, the male-specific neurons consist of different physiological categories being specifically tuned to the behaviorally relevant compounds, including pheromone neurons, ensuring the male sexual response and neurons responsible for interspecific antagonism, here termed non-pheromone neurons.

### 3.1. The Pheromone Components Are Generally Recognized by Two Neuron Types Being Separated in Distinct Sensilla

Based on single cell recordings from antennal sensilla trichodea of various heliothine males, a population of pheromone-specific neurons has been found in all species [27,28,29,30,31,32,33,34,35,36], consisting of two physiological types that respond selectively to each of the pheromone components of a particular species. In addition, OSNs specifically tuned to substances produced by sympatric heterospecific females have been identified in most of the species studied. For example, males of *H. vires*c*ens* possess two types of pheromone OSNs tuned to Z11-16:AL and Z9-14:AL, respectively, whereas two additional neuron populations are tuned to compounds produced/used by sympatric species, one to Z11-16:AC and the other to Z11-16:OH [28,29,32,33,37]. In males of the Oriental tobacco moth, *H. assulta*, two types of pheromone neurons tuned to Z9-16:AL and Z11-16:AL, respectively, have been identified [31], plus a population of Z9-14:AL-responding neurons that carry interspecific information.

Interestingly, the total repertoire of male-specific neurons, usually including two pheromone types and one or two non-pheromone types in each species, form a particular pattern with respect to compartmentalization or separation within distinct sensilla. As previously pointed out by Berg *et al*. [38], all possible combinations of pairing these neuron types within the same sensillum have been reported in the heliothine species studied, except for one: namely, that of the co-localization of the neuron tuned to the major pheromone component alongside a neuron tuned to any minor component. In *H. subflexa*, one type of sensillum does have paired neurons tuned to the two secondary components (Table 1) (Figure 1). Thus, similar to several noctuid moths using binary pheromone blends, as, for example, *Spodoptera littoralis* [39], co-localization of the neurons tuned to the two pheromone components is usually not found in heliothine species. The principle of separation allows an unequal abundance of the two neuron types; evidently an expedient system, since all species have a major neuron population tuned to the primary pheromone component and a minor one tuned to the second component. The option that such an arrangement may actually be advantageous for apprehending the species-specific pheromone blend in an optimal way should not be excluded, particularly when taking into account recent findings reporting that one OSN may inhibit a neighboring neuron housed within the same sensillum [40]. However, because some moths do possess pheromone neurons being co-localized within the same sensilla, as, for example, the pyralid species *Ostrinia nubilalis* [41], there is obviously more than one alternative for an optimal organization of the concerned neuron types. Electrophysiological investigations performed on another species possessing paired pheromone neurons, *Argyrotaenia velutinana*, have demonstrated that the ratio of the critical pheromone components is well represented in this system, as well, via neurons displaying high fidelity in their responses to the individual components [42]. Furthermore, previous studies have even reported that an arrangement of co-localized neurons improves the ability of a moth [43] and a bark beetle [44] to discriminate closely-separated odor sources. The paired neurons involved here were not two pheromone-sensitive neurons, however.

### 3.2. Interspecific Antagonism Is Ensured via Sensory Neurons Often Being Paired with Pheromone Neurons

The presence of OSNs for pheromone components of a related, neighboring species, having an antagonistic effect on attraction, has been demonstrated in several heliothine moths (Table 1). Thus, one particular female-produced compound is sometimes recognized by similar neuron types being present in sympatric males. These functionally different neuron populations, serving as pheromone and non-pheromone neurons, respectively, may even possess similar physiological characteristics; one group of Z9-14:AL-sensitive neurons identified in *H. zea* is in fact reported to display identical response properties to those of a corresponding neuron population in *H. virescens*, even though they are correlated with the mediation of interspecific information in the former species and pheromone information in the latter [28,30,32,33,34].

Sensory neurons involved in behavioral antagonism often differ from the highly selective pheromone neurons by responding to several female-produced compounds. In *H. assulta*, for example, the non-pheromone Z9-14:AL neurons are strongly activated by a second best stimulus, *i.e.*, *cis*-9-hexadecenol (Z9-16:OH) [31]. Behavioral studies have reported that both compounds, Z9-14:AL and Z9-16:OH, decrease the male attraction when added to the pheromone blend of the conspecific female [11,24]. Furthermore, males of *H. zea* possess one non-pheromone neuron type responding with similar strength to Z11-16:OH and Z11-16:AC [34], both of which are reported to suppress the male sexual behavior [19,20,21,22,23]. The arrangement of non-pheromone neurons displaying broader response profiles than those tuned to pheromones may be advantageous, since this enables species-specific isolation concerning several sympatric heterospecific females via the same neuron population. Corresponding findings have been reported in other moth species [45]. Having pointed out the general trend, it should be mentioned that interspecific signal information being detected by distinct highly specific neuron types has been reported, as well; as previously stated, two non-pheromone neurons have been found in *H. virescens,* responding to Z11-16:AC and Z11-16:OH, respectively [29,37].

Neurons tuned to interspecific signals are often co-localized in the same sensillum type along with a pheromone-specific neuron. In *H. assulta*, for example, one unusually large population of neurons tuned to a behavioral antagonist is present due to the fact that they are located within sensilla housing the neuron tuned to the primary pheromone component [38]. In *H. zea* and *H. armigera*, on the other hand, non-pheromone neurons constitute minor populations, since they are co-located with neurons responding to the second pheromone component [46]. Actually, behavioral tests performed on *H. zea* have demonstrated the insect’s exquisite ability of discriminating odor sources, including stimuli activating these co-localized neuron categories. As reported by Baker, the males can distinguish completely coincident strands of the pheromone and the interspecific signal occurring from sources placed next to each other at a distance of only 1 mm [43]. Taking into account the recent finding demonstrating non-synaptic inhibition between neurons being paired within the same sensillum [40], a co-localization, including neuron types being associated with opposite behavioral responses, seems indeed appropriate.

### 3.3. Comparison of Physiological Sensillum Types across the Various Species

Because the grouping and/or separation of neurons within trichoid sensilla share certain characteristics across the various species, a general naming of the sensilla has been established. The naming originates from three sensillum types housing the male-specific neurons identified in *H. zea*, termed the A-, B- and C-type sensillum (Figure 1) [34].

The A-type sensillum houses a Z11-16:AL-responding neuron in all species studied. The tuning profile and sensitivity of these neurons, which are the most abundant in all species, except for *H. assulta*, seem to be consistent across the various species. The neuron tuned to Z11-16:AL is sometimes reported to be co-localized with another non-identified neuron displaying distinctly lower spontaneous activity [37,47]. In *H. virescens*, molecular studies have identified a putative odorant receptor (OR), the so-called HR11, for the as-yet physiologically uncharacterized neuron in A-type sensilla. These neurons are arranged in a paired pattern together with neurons expressing HR13, which has been shown to be most sensitive to the ligand Z11-16:AL [48].

The B-type sensillum contains a neuron responding to Z9-14:AL/Z9-16AL. This neuron type possesses similar physiological properties in both *H. zea* and *H. virescens* in spite of being functionally distinct. Thus, as previously mentioned, the neuron responds best to Z9-14:AL in both species [27,32,34]. In *H. subflexa*, however, the B-type sensillum is slightly different, because it houses a neuron responding considerably more strongly to Z9-16:AL than to Z9-14:AL [29]. Type-B sensilla, which have not yet been found in *H. assulta* and *H. armigera* [38], may be present in these two species, as well. Similar to the A-type sensilla, the B-type of *H. virescens*, *H. zea* and *H. subflexa* is reported to house a second neuron whose physiology has yet to be characterized, because none of the tested odorants have been shown to activate it [35,47].

The third category of sensillum, the C-type, contains two co-localized neurons, one displaying larger amplitude spikes than the other. Apart from the general principle implying that at least one of the adjacently located neurons always responds to an interspecific signal, the C type sensilla vary to a great extent across the species studied. In the Oriental species, *H. assulta*, for example, it consists of one large-spiking neuron responding to Z9-16:AL, the major pheromone component and one small-spiking neuron responding to the two antagonists, Z9-14:AL/Z9-16:OH [38]. In *H. zea*, on the other hand, it houses one large-spiking neuron tuned to Z9-16:AL/Z9-14:AL and one small-spiking tuned to Z11-16:AC/Z11-16:OH acting as behavioral antagonists [34,46]. Male-specific neurons tuned to interspecific signals are involved in all possible combinations of pairing/separation, including that of grouping two non-pheromone neurons within the same sensillum, as exemplified by the C-type sensilla in *H. virescens* housing two neurons responding to Z11-16:OH and Z11-16:AC, respectively [29,37].

A certain topographical distribution of the three types of sensilla seems to be present, as the A-type is frequently found along the regions located adjacent to the scaled part of the antenna, whereas the B- and C-type sensilla often occur among the somewhat shorter sensilla trichodea located in the mid-ventral region [27,49].

### 3.4. Hybrids as Models for Studying Basic Determinants Underlying Speciation

Successful hybridization of closely related heliothine species in the laboratory has provided the opportunity of exploring putative evolutionary aspects linked to the male-specific olfactory system [50,51]. Since *H. armigera* and *H. assulta* possess similar types of pheromone neurons, but in opposite ratios, hybrids of these sibling species are particularly interesting objects for such studies. Both behavioral and electrophysiological investigations have shown that such hybrids are significantly more similar to *H. armigera* than to *H. assulta*, indicating a dominance of the former species’ genes [52]. Furthermore, hybrids of *H. virescens* and *H. subflexa* have been models in behavioral and electrophysiological studies, here demonstrating ascendancy for the features characterizing *H. subflexa* [53,54,55]; though these hybrids also showed behavioral responses to blends containing high doses of Z9-14:AL, which is the second pheromone component of *H. virescens* [54]. Correspondingly, single-cell recordings demonstrated that OSNs tuned to Z9-16:AL in the hybrids displayed a relatively high sensitivity to Z9-14:AL, as well [53]. Whether the shifted response pattern is caused by the increase or decrease of particular pheromone receptors is an open question.

### 3.5. Molecular Basis for Detection of the Female-Produced Compounds

Based on the comprehensive knowledge about the male-specific neurons in heliothine moths, pointing to general principles regarding categories, as well as species-specific features of their response properties, it is particularly interesting to compare the physiological characteristics of the relevant receptors. In recent years, numerous investigations have been performed in several heliothine species aiming to explore the molecular basis for the detection of the relevant female-produced substances. Odorant receptors expressed in neurons confined to the male-specific sensilla of *H. virescens*, discovered by Krieger and colleagues one decade ago, were actually among the first pheromone receptor candidates identified in insects [56]. At present, six male-specific receptors from the investigated species have been cloned and physiologically characterized [56,57,58]. In addition, a similar number of pheromone receptor candidates has been identified in *H. assulta* and *H. armigera* [59,60], six of which were recently physiologically described in *H. armigera* and three in *H. assulta* [61,62]. For simplicity, we have chosen to name the six receptors identified in *H. virescens* HvirOR6, HvirOR11 and HvirOR13–HvirOR16 (originally termed HR6, HR11 and HR13–HR16), as the latter naming system corresponds with that used for the two *Helicoverpa* species.

In general, the receptors identified in the various species show binding properties correlating with physiological data from electrophysiological recordings performed on the OSNs. For example, in accordance with the physiologically identical Z11-16:AL-neurons housed by the A-type sensilla, the three species express OR13 receptors displaying similar properties by binding specifically to Z11-16:AL [57,58,61,62,63]. Furthermore, in line with the partly more diverse neuron types housed in the other sensillum types, the remaining receptors identified seem to a greater extent to vary across the species. One example is the OR16 receptor; both in *H. virescens* and *H. armigera*, the relevant orthologs, HvirOR16 and HarmOR16, bind to Z11-16:OH [58,61,62]. In *H. assulta*, however, Z9-14:AL is reported to be the ligand for HassOR16 [62]. Previous electrophysiological studies have shown that Z11-16:OH activates one of the paired neurons housed by the C-type sensilla in both *H. virescens* [29,37] and *H. armigera* [64], whereas *H. assulta* possesses no neuron tuned to this alcohol [31]. Interestingly, HassOR16, showing 93% amino acid identity with HarmOR16, seems to have evolved distinct characteristics for adapting to the particular requirements of the species [62]. This example demonstrates that genes sharing relatively similar amino acid sequences may in fact encode receptors being specialized for dissimilar ligands. In spite of different binding properties characterizing the OR16 orthologs across the species, one general feature seems to be that this receptor is expressed in a non-pheromone neuron housed by the C-type sensillum, however.

In addition to the pheromone receptors, another essential category of proteins being present at the peripheral part of the male-specific olfactory pathway is the pheromone binding proteins (PBPs). During the last decade, PBP genes from *H. virescens*, *H. zea*, *H. armigera* and *H. assulta* have been cloned and identified [65,66,67,68,69,70,71,72]. In particular, the presence of a pheromone-binding protein, HvirPBP2, playing an essential role for detection of the primary pheromone component, Z11-16:AL, has been reported in *H. virescens* males [57].

## 4. Central Processing of Information about Pheromones and Interspecific Signals

Like in other moths, the axons of the male-specific OSNs of heliothine species project directly to the primary olfactory center of the brain. Here, in the antennal lobe, they target a small number of enlarged glomeruli, called the macroglomerular complex (MGC), located dorsally in the antennal lobe, at the entrance of the antennal nerve (Figure 1B) [73]. An additional assembly of so-called ordinary glomeruli, including approximately 60 units in heliothine species, seems to be equivalent to the ordinary glomeruli described in females [74,75,76]. These glomeruli receive input from OSNs detecting mainly florals and plant volatiles [77,78]. An arrangement, including two female-specific glomeruli, has been reported in *H. virescens* [74,78].

### 4.1. Anatomy and Physiology of the Male-Specific MGC in a Comparative Perspective

Common for all MGCs of heliothine species studied so far is an arrangement of one large, centrally-located unit, the cumulus, being surrounded by two or three smaller glomeruli. These satellite glomeruli are named after their locations with respect to the cumulus (Figure 1C–F). In species of *Helicoverpa*, the total number of MGC-units is three [74,75,79,80] and in species of *Heliothis*, four [37,74,80,81].

In full agreement with the so-called principle of chemotopy, the various physiological types of male-specific OSNs project to distinct and identifiable MGC units in the antennal lobe [33,35,37,38,46]. Generally, the cumulus is shown to process information about the major pheromone component, whereas the satellite glomeruli are associated with the second pheromone component and interspecific signals (Figure 1C–F). Thus, in all of the species studied, except for *H. assulta*, a considerable amount of Z11-16:AL-responding OSNs and antennal-lobe projection neurons have been described, all of which extend neural processes in the cumulus [33,35,37,46,79,80,81,82]. In *H. assulta*, using Z11-16:AL as the critical secondary pheromone component, the corresponding neurons are connected to one of the satellite glomeruli, whereas the cumulus is innervated by neurons tuned to Z9-16:AL, serving as the primary pheromone constituent of this species [38,83].

The uniqueness of *H. assulta* is, in fact, apparent just by considering the anatomical arrangement of the MGC units. Thus, different from the other *Helicoverpa* species studied so far, *i.e.*, *H. armigera* and *H. zea*, possessing two dorsomedial satellite glomeruli, *H. assulta* has one dorsomedial and one ventral glomerulus surrounding the cumulus (Figure 1G) [38,74]. The three species utilize the same principal pheromone constituents, Z11-16:AL and Z9-16:AL, but in *H. assulta*, the compounds are present in a ratio being opposite of that of the others. As previously suggested by Zhao and Berg [83], the opportunity of comparing the physiological tuning of the MGC-units in these species, particularly in *H. armigera* and *H. assulta* inhabiting the same geographical region, may reflect how dynamic transformations of brain structures essential for speciation have evolved. For example, a change including an increased size of the small unit located ventrally in the MGC of *H. assulta*, tuned to Z11-16:AL, on the cost of the large unit tuned to Z9-16:AL, could possibly form an MGC similar to that of *H. armigera* (Figure 1).

Species-specific tuning properties of particular units have also been reported in the *Heliothis* species, *H. virescens* and *H. subflexa*, which possess four identically-arranged MGC glomeruli. Whereas the cumulus in these moths is associated with the same compound, Z11-16:AL, the tuning of the dorsomedial unit differs in line with the species’ use of dissimilar constituents as the second pheromone component, being Z9-14:AL in *H. virescens* and Z9-16:AL *in H. subflexa* [81]. Furthermore, the two smaller MGC-units located ventrally are reported to have switched their physiological tuning in the two species, possibly entailing a shift in the functional significance of one particular glomerulus (based on the assumption that Z11-16:OH is an essential pheromone component in *H. subflexa*) [81].

Regarding Z9-14:AL, being used as the secondary pheromone component in *H. virescens* and as an interspecific signal in *H. zea*, *H. assulta* and *H. armigera,* the information about the compound is, without exception, being processed in one dorsomedially-located glomerulus [80,81,83]. In the American *H. zea*, this MGC-unit, the anterior dorsomedial glomerulus, is reported to process information about another interspecific signal, as well; thus, branches of both Z9-14:AL- and Z11-16:AC-tuned second order neurons arborizing in this glomerulus have been found [80]. Correspondingly, the MGC-unit is in fact found to receive input from two categories of OSNs, *i.e.*, Z9-14:AL- and Z11-16:AC-responding neurons located in B- and C-type sensilla, respectively [46], an arrangement apparently deviating from the one-receptor-one-glomerulus rule.

### 4.2. The Anatomical Separation of Pheromone and Plant Odor Signals Is Maintained in the Antennal-Lobe Projection Neurons

Like in other moth species, the glomerular network of heliothines includes two main categories of antennal-lobe neurons in addition to the sensory axon terminals, *i.e.*, projection neurons carrying the olfactory information to two main integration centers in the protocerebrum, the calyces of the mushroom bodies and the lateral horn, plus local interneurons being confined to the antennal lobe [83,84,85]. For terminology, see Ito *et al*. [86]. In addition to mapping the target regions of male-specific OSNs, the tuning of individual MGC units to particular odor compounds is described via tracing of physiologically-characterized projection neurons [37,79,80,81,82,83] and calcium imaging experiments [36,87]. The total population of projection neurons makes up three main antennal-lobe tracts (ALTs), termed the medial ALT (mALT), the medio-lateral ALT (mlALT) and the lateral ALT (lALT) [85,86,88]. Pheromone information is reported to be carried in all three tracts [88,89]. Former studies, including mainly uniglomerular projection neurons passing in the mALT, have shown that pheromone and plant odor information is separated, corresponding to the arrangement at the peripheral level [37,79,80,81,82,83]. So far, none of the male-specific projection neurons characterized, including multiglomerular pheromone-processing neurons, have been found to innervate any glomeruli outside the MGC. However, based on studies from several other moth species showing that pheromone responses are modulated by plant odors, a corresponding interplay presumably takes place in heliothine moths, as well [90,91,92]. Local interneurons connecting the MGC with ordinary glomeruli have been found in several moth species, heliothine moths included [93,94,95]. In *H. virescens*, it has actually been reported that the presence of plant odorants decreases the response at the site of the OR13 receptor when added to the relevant pheromone [96]. Electrophysiological recordings from OSNs tuned to the relevant pheromone compound have demonstrated both increased and decreased responses when adding plant odors to the key compound, however [97,98].

### 4.3. Most Male-Specific Projection Neurons Are Confined to the mALT Display Relatively Narrowly-Tuned Response Profiles

The input to and output from the MGC-units in heliothine moths seems, to a great extent, to match, meaning that the uniglomerular projection neurons display response patterns in line with the odotopic mapping of the distinct glomeruli. The arrangement of anatomically-separated systems for information about pheromones and interspecific signals at this level is particularly well documented [79,80,82,83]. Keeping the pheromone information separated from the inter-specific signal information seems sensible, based on the opposite behavioral responses induced by the different signals. An arrangement of maintaining a separation of the information about the two pheromone components at the present level may not seem correspondingly reasonable, however, based on their similar role in ensuring the male sexual response. Nevertheless, the majority of pheromone projection neurons identified in heliothine moths to date is actually found to respond selectively to one component [79,80,81,83], meaning that the information about the two components, to a great extent, is carried in distinct second order neurons. Having pointed out the most common organization, it should be emphasized that there is also an essential element of combinatorial coding deviating from the arrangement of specifically tuned channels, as found in the periphery. In *H. virescens*, for example, uniglomerular projection neurons demonstrating synergistic responses to the pheromone components, Z11-16:AL and Z9-14:AL, have been reported [37,80]. Furthermore, in *H. assulta,* particular projection neurons innervating one of the satellite glomeruli and being excited by antennal stimulation with the interspecific signal, Z9-14:AL, are inhibited by the major pheromone component [83].

### 4.4. Odor Signals Associated with Distinct Behaviors Are Differently Represented in the Lateral Horn

**Figure 2:**
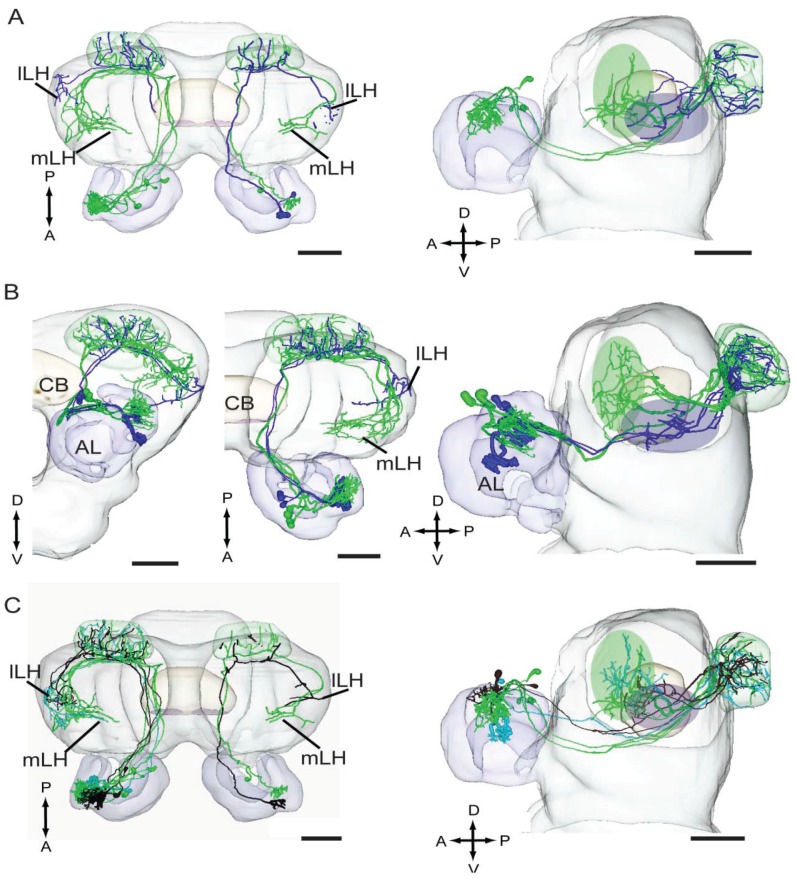
Digital reconstructions of individual medial-tract projection neurons obtained from males of *Heliothis assulta* and *Heliothis virescens,* which have been integrated into standard brain atlases of the respective species, adapted from Zhao *et al*. [89]. (**A**,**B**) Both in *H. assulta* and *H. virescens*, the projection neurons tuned to plant odors (blue) and pheromones (green) terminate in non-overlapping regions of the lateral horn (LH). (**C**) In *H. assulta*, the two projection neuron categories carrying pheromone information (green and turquoise) target a completely overlapping region of the LH, whereas the neurons tuned to the interspecific signal (black) target a smaller and partly differently located area. lLH, lateral part of the lateral horn; mLH, medial part of the lateral horn; CB, central body; AL, antennal lobe; P, posterior; A, anterior; D, dorsal; V, ventral. Scale bars: 100 µm.

As compared to the extensive knowledge about the well-organized MGC of heliothine moths, demonstrating a non-overlapping representation of the biologically relevant stimuli, less is known about the neural arrangements residing at the following synaptic level, *i.e.*, the calyces of the mushroom bodies and the lateral horn. In all heliothine species studied, the male-specific mALT-neurons are reported to terminate in a particular area located medially in the lateral horn [79,80,81,82,83]. Recent findings have demonstrated that this particular region is distinct from that targeted by plant odor neuron (Figure 2A,B) [89]. Corresponding findings have been reported in other moth species [88,99]. The principle of separating the representation of odor stimuli according to biological significance in the lateral horn seems to apply not only to plant odors and social cues, but also to conspecific and interspecific signals. Thus, in *H. assulta*, it has been found that male-specific projection neurons tuned to each of the two pheromone components target an overlapping region in the lateral horn, whereas projection neurons carry interspecific signal information terminate in a smaller and partly distinct region (Figure 2C) [89]. Generally, the new data indicate that the male-specific terminals in the lateral horn, to a certain degree, are spatially organized, not according to the identity of the associated odor component or the MGC glomerulus they are linked to, but according to their functional significance.

### 4.5. Modulatory Input to the Antennal Lobe

A third and relatively small category of antennal-lobe neural elements is the centrifugal neurons having their dendritic branches in various brain regions and an axon projecting into the antennal lobe, often innervating all glomeruli. Centrifugal antennal-lobe neurons identified in heliothine moths include the so-called serotonin-immunoreactive antennal-lobe neuron [100] and a multimodal neuron type responding to sound and odor [101]. Both neuron categories innervate seemingly all antennal-lobe glomeruli, including the MGC, indicating that other factors than odor may modulate the pheromone information in the antennal lobe. Particularly intriguing is the multimodal neuron type, described for the first time in two geographically-isolated heliothine species, *i.e.*, *H. virescens* and *H. armigera* [101]. This ipsilateral neuron type has fine processes in the dorsomedial region of the protocerebrum and extensive neuronal branches with blebby terminals in all glomeruli of the antennal lobe. In both species, the neuron was excited by transient sound pulses, including the ultrasound-like echolocation calls of a bat. These findings are certainly interesting, especially when considering the number of studies having reported that moths frequently adjust their behavior based on sensory input through two modalities, odor and sound, in order to find a mate and to avoid hunting bats [102,103,104,105,106,107].

## 5. Conclusions

The male-specific olfactory system of moths offers the opportunity of investigating a relatively simple neural network of considerable importance for reproductive success. Heliothine moths are especially interesting due to the fact that they use female-produced signals for communication not only interspecifically but also across sympatric species; thus, a few odour stimuli, each being represented in an easily identifiable glomerulus in the olfactory centre of the male brain, are linked to one of two well defined behavioural responses, attraction or interruption of attraction. Encoding mechanisms characterizing the male-specific pathway of heliothine moths are thoroughly studied both at the peripheral and central level. In general, the behaviourally relevant odours are recognized by sensory neurons that to a large extent respond selectively to each component. Such a ‘labeled line’ system may in fact be a suitable arrangement for processing information about stimuli that are basically discontinuous. Like in other insects, the odour stimuli are distinctly represented in a highly spatial order in the primary olfactory centre whereas a fundamentally different pattern, including a considerable extent of overlap and yet a spatial arrangement of projections carrying odour signals associated with different behaviours, appears in the LH. In heliothine moths, this arrangement includes information about pheromones, interspecific signals, and general odorants. The identification of several antennal-lobe centrifugal neurons in this sub-family indicates the importance of modulatory input to the olfactory pathway. In general, the nervous system of heliothine moths offers the opportunity of revealing not only functional circuits underlying so-called stereo-typed response patterns but also more complex networks linked to learning and behavioural adaptations.
